# *In silico* analysis reveals EP300 as a panCancer inhibitor of anti-tumor immune response via metabolic modulation

**DOI:** 10.1038/s41598-020-66329-7

**Published:** 2020-06-10

**Authors:** Rosemarie Krupar, Christian Watermann, Christian Idel, Julika Ribbat-Idel, Anne Offermann, Helen Pasternack, Jutta Kirfel, Andrew G. Sikora, Sven Perner

**Affiliations:** 1 Pathology of the Research Center Borstel, Leibniz Lung Center, Borstel, Germany; 2 Pathology of the University Hospital Schleswig-Holstein, Campus Luebeck, Luebeck, Germany; 3 Department of Otorhinolaryngology, University Hospital Schleswig-Holstein, Luebeck, Germany; 4 Department of Otorhinolarygology – Head and Neck Surgery, Baylor College of Medicine, Houston, USA

**Keywords:** Tumour immunology, Cancer genomics, Translational research, Predictive markers, Molecular medicine

## Abstract

The tumor immune microenvironment (TIME) of head and neck squamous cell carcinomas (HNSCC) and other solid malignancies is a key determinant of therapy response and prognosis. Among other factors, it is shaped by the tumor mutational burden and defects in DNA repair enzymes. Based on the TCGA database we aimed to define specific, altered genes associated with different TIME types, which might represent new predictive markers or targets for immuno-therapeutic approaches. The HNSCC cohort of the TCGA database was used to define 3 TIME types (immune-activated, immune-suppressed, immune-absent) according to expression of immune-related genes. Mutation frequencies were correlated to the 3 TIME types. Overall survival was best in the immune-activated group. 9 genes were significantly differentially mutated in the 3 TIME types with strongest differences for *TP53* and the histone-acetyltransferase *EP300*. Mutations in *EP300* correlated with an immune-activated TIME. In panCancer analyses anti-tumor immune activity was increased in *EP300* mutated esophageal, stomach and prostate cancers. Downregulation of EP300 gene expression was associated with higher anti-tumor immunity in most solid malignancies. Since EP300 is a promoter of glycolysis, which negatively affects anti-tumor immune response, we analyzed the association of EP300 with tumor metabolism. PanCancer tumor metabolism was strongly shifted towards oxidative phosphorylation in EP300 downregulated tumors. *In silico* analyses of of publicly available *in vitro* data showed a decrease of glycolysis-associated genes after treatment with the EP300 inhibitor C646. Our study reveals associations of specific gene alterations with different TIME types. In detail, we defined EP300 as a panCancer inhibitor of the TIME most likely via metabolic modulation. In this context EP300 represents a promising predictive biomarker and an immuno-therapeutic target.

## Introduction

The tumor immune microenvironment (TIME) has emerged as a critical factor determining prognosis and therapy response across most solid tumors. In immunogenic tumors, such as breast and colon cancers or non-small cell lung cancers, the quantity and quality of tumor infiltrating lymphocytes has prognostic significance comparable to the TNM-classification^1^. The TIME is highly variable across different cancer entities and cancer subtypes as well as over the course of cancer progression and treatment^2,3^. Nevertheless, solid tumors can generally be classified into different categories according to their specific TIME profiles. We can distinguish at least three different TIME types in solid tumors, considering their prognostic impact and the quantity and quality of tumor infiltrating leukocytes^2,4^:

I. Tumors with a predominantly anti-tumor TIME, characterized, among other factors, by a high number of CD8** +** cytotoxic T cells (CTLs) and other lymphocytes such as CD4 T helper cells, natural killer (NK) cells, regulatory T cells (Tregs), B lymphocytes, plasmocytoid dendritic cells as well as elevated levels of granzyme and perforin.

II. Tumors with a predominantly pro-tumor TIME, characterized by few infiltrating lymphocytes and a high number of infiltrating myeloid cells, in particular M2 macrophages and myeloid derived suppressor cells (MDSC).

III. Tumors with an immune-absent TIME, characterized by an overall absence of tumor infiltrating leukocytes.

Factors regulating the TIME composition are not understood in a detailed fashion. The attraction of tumor infiltrating myeloid cells is considered to depend on the production of chemokines such as VEGF, GM-CSF and TGF-β by tumor immune and stromal cells^5^. The amount of tumor infiltrating CD8 CTLs and their cytolytic activity, in contrast, is primarily influenced by the immunogenicity of the tumor. Tumor cells express antigens, which can induce a targeted anti-tumor immune response, when presented to T cells via major histocompatibility complexes (MHC) by antigen presenting cells (APC). Several mechanisms can lead to the formation of tumor antigens. One mechanism is the expression of germ-line genes (“cancer-testis antigens”). In healthy persons these are only expressed by male germ line cells and are usually not presented by MHC to induce T cell tolerance during thymic maturation. Another source of tumor antigens are oncogenic viruses, such as Human Papilloma Virus (HPV) or Epstein-Barr Virus (EBV), which can encode antigenic peptides. Over the last years tumor mutations (mostly non-synonymous point mutations) have been brought into focus as another source of tumor antigens. These lead to modified peptide sequences (“mutational neoantigens”), which can induce a T cell response, when presented by MHC^6^. The amount of non-synonymous mutations (tumor mutational burden/TMB) has been shown to correlate with anti-tumor immunity, outcome and response to treatment with immune checkpoint inhibitors^4,7^. Further investigations have elaborated the role of a functional antigen presenting machinery, to make optimal use of the TMB for an anti-tumor immune response^8^. Tobacco- and alcohol-induced squamous cell carcinomas of the head and neck, the esophagus and the lung are among the cancers with a high mutational load^9^. A high TMB and high CD8 CTL infiltrates predict particularly in head and neck squamous cell carcinoma (HNSCC) a benefit from PDL-1 inhibitor treatment^10^. In this study we aimed to discover specific mutations associated with specific TIME profiles and therefore constituting predictors for response to immune checkpoint inhibition or as potential adjuncts to improve efficacy of current immunotherapeutic agents.

## Materials and Methods

### The Cancer Genome Atlas (TCGA) database analysis

TCGA data bank analysis was performed on the cBioPortal for Cancer Genomics website (http://www.cbioportal.org) with access to clinical data, gene mutations and gene expression levels. All analyses were based on TCGA data provided by cBioPortal until December 2018. HPV status were deduced from a previous extensive characterization of the TCGA database by Rooney et *al*.^4^.

### Characterization of tumor immune microenvironment (TIME) types

**Figure 1:**
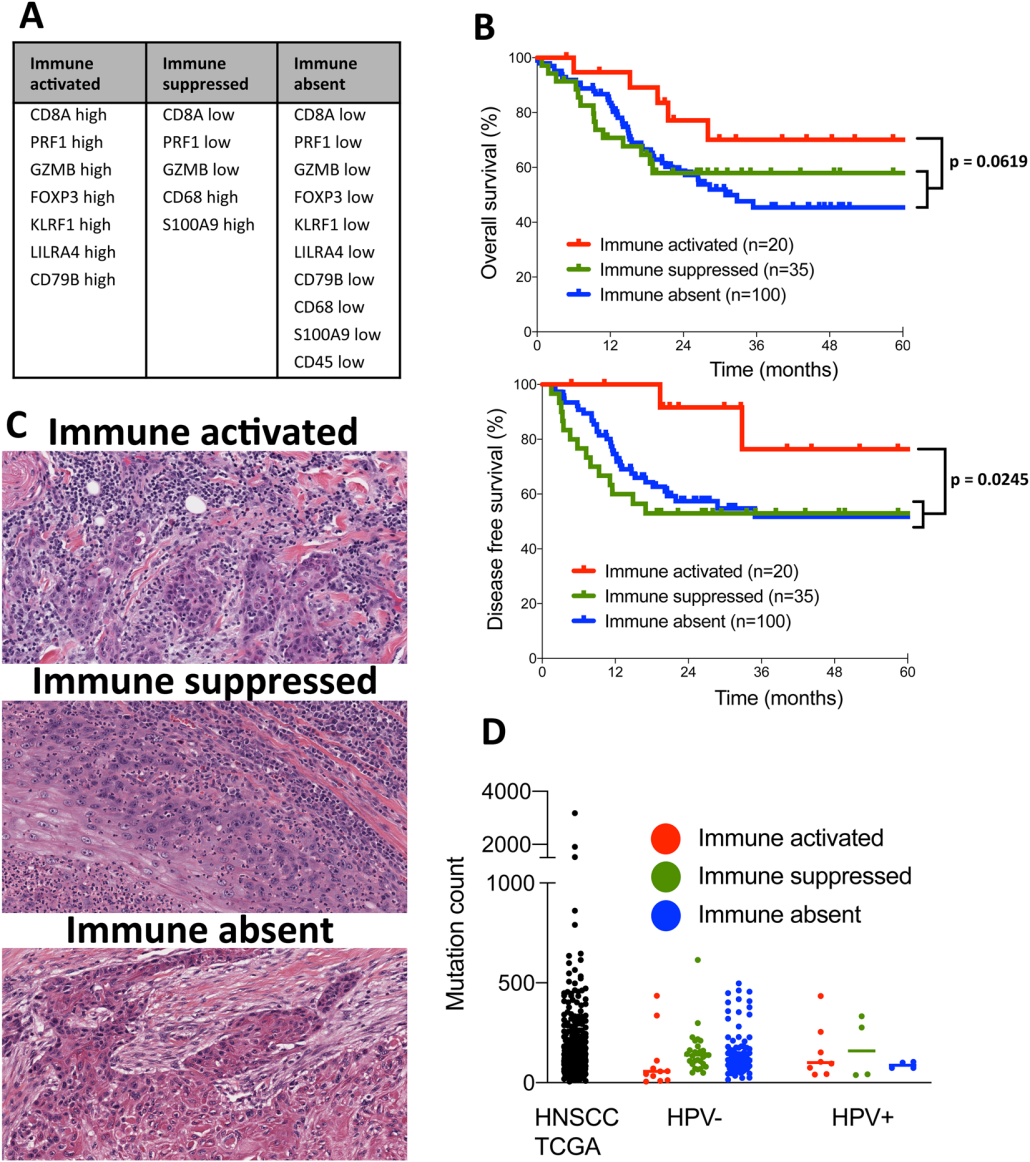
Gene expression-based tumor immune microenvironment (TIME) subtypes in HNSCC are clinically relevant and reflect histological phenotypes, but do not correlate with tumor mutational burden. (**A**) List of genes defining different TIME subtypes. (**B**) Patient subgroups with immune-activated, immune-suppressed and immune-absent TIMEs with best overall and disease free survival for immune-activated TIME patients. (**C**) Histological pictures of one representative immune-activated tumor with strong lymphocytic immune cell infiltrate (patient TCGA-CV-A468), one immune-suppressed tumor with a background of eosinophils and neutrophils (patient TCGA-BA-A6DA) and one immune-absent tumor without any immunocytes (patient TCGA-CN-5376). (**D**) Non-synonymous mutation counts for all HNSCC TCGA patients and for the three TIME subtypes subdived by HPV status with no signficant differenes between the groups.

Molecular TIME types were defined according to mean expression levels of transcriptomic cell markers (z-Score = 0), as outlined in Fig. 1A. Genes used for transcriptomic cell type markers were selected from the TCGA database study by Rooney et *al*.^4^. A focus was put on genes with established immunohistochemical markers in diagnostic pathology work-up (CD45, CD8A, FOXP3, CD79A, CD68, Granzyme, Perforin). Based on an additional study, S100A9 was included as a gene expression marker for myeloid derived suppressor cells^11^. Correlation of molecular TIME types with histological tumor appearances was performed using the digital slide archive (DSA, https://cancer.digitalslidearchive.org), which provides scanned Hematoxilin- and Eosin- (H&E-) stained tumor tissue slides of the TCGA cohorts^12^. One board-certified pathologist (R. K.), experienced in head and neck pathology, performed slide evaluations. The following criteria were applied to assign the histological tumor slides to the 3 TIME types:

- Immune-activated: Rich lymphoplasmocytic infiltrate in the desmoplastic peritumoral stroma and at least occasional presence of tumor infiltrating lymphocytes (TILs)

- Immune-suppressed: Presence of neutrophils, eosinophils, macrophages or mast cells in the desmoplastic peritumoral stroma and at least occasional presence of tumor infiltrating neutrophils, eosinophils, macrophages or mast cells

- Immune-absent: Few immunocytes in the desmoplastic peritumoral stroma and mostly absence of tumor infiltrating immunocytes.

Cytolytic activity was used as a measurement of anti-tumor immune response. Analogous to Rooney *et al*. cytolytic activity was based on simultaneous upregulation of GZMB (granzyme) and PFN1 (perforin) according to mean gene expression levels (z-Score = 0). EP300 upregulation was determined according to mean EP300 expression with a z-Score = 1.

The tumor immune subtypes C1- C6 were downloaded from the Supplementary Files of the publication by Thorsson *et al*. for further correlations^13^.

### Annotation of mean total mutation count and specific non-synonymous gene mutations in the TCGA database

The total number of non-synonymous gene mutations per HNSCC patient were accessed via cBioPortal, in order to compare the mean total mutation count in different TIME types. In the next step, only genes with a mutation frequency of at least 10x in the HNSCC TCGA cohort were selected for further analysis. This represents a mutation frequency of 1.95% and could include several non-synonymous mutations in one gene of one patient as well as one mutation in one gene of several different patients.

### Metabolic characterization of the TCGA database

Metabolic characterizations of tumors were executed via analyses of gene expression levels involved in energy metabolism. The Glucose Metabolism RT^2^ Profiler PCR array and the Mitochondrial Energy Metabolism RT^2^ Profiler PCR array by Qiagen (Hilden, Germany) served as configuration standards to compile lists of relevant genes for metabolism studies. Based on genes represented in these arrays a glycolysis-dependent and an oxidative phosphorylation- (OXPHOS-) dependent tumor metabolism were defined (z-Score = 0). Based on our own previous studies the glucose transporter 1 (GLUT1) as well as lactate dehydrogenase A and B (LDHA, LDHB) were added to the glycolysis-dominant gene list^14,15^.

In summary the glycolysis-dominant list consisted of 24 enzymes and transporters involved in glucose and pyruvate/lactate metabolism: ALDOA, ALDOB, ALDOC, BPGM, ENO1, ENO2, ENO3, GALM, GCK, GPI, HK2, HK3, PFKL, PGAM2, PGK1, PGK2, PGM1, PGM2, PGM3, PKLR, TPI1, GLUT1, LDHA, LDHB.

The OXPHOS-dominant list was composed of 31 mitochondrial proteins necessary for the formation of complex I – V of the respiratory chain: ATP12A, ATP5A1, ATP5F1, ATP5G3, ATP5J, ATP5O, ATP6V1C2, LHPP, OXA1L, PPA1, COX4I1, COX5B, COX6B1, COX7A2, COX8A, BCS1L, UQCRC1, UQCRH, SDHA, SDHC, NDUFA1, NDUFA2, NDUFA5, NDUFA8, NDUFB2, NDUFB5, NDUFB8, NDUFC2, NDUFS3, NDUFS6, NDUFV1.

The Visualization of metabolic tumor phenotypes was achieved by the word cloud generator www.wortwolken.com.

### Metabolic characterization of cell lines after EP300 inhibition

The L1000 Connectivity Map data (L1000 CMap), provided by the Broad Institute, was used to study the effects of EP300 inhibition on cell metabolism in different cell lines^16^. The analyses were performed with Genevestigator (Nebion AG, Zurich, Switzerland). The following experiments of the L1000 CMap were included into the study: 1. Controls: Cell lines treated with DMSO for 24 hours (10 replicates for each cell line). 2. Cell lines treated with 10 μM C646 for 24 hours (3–6 replicates for each cell line). Matched data was available for 8 different cell lines (A375, A549, HA1E, HCC515, HEPG2, HT29, MCF7, PC3). EP300 mutation and baseline expression status was extracted from cBioPortal. The glycolysis-dominant list of genes was expanded by an addition of 30 glycolysis genes provided by Genevestigator. This led to a total of 52 genes with available L1000 CMap gene expression data to characterize a glycolysis-associated metabolism. 112 OXPHOS-related genes provided by Genevestigator were added to the OXPHOS-dominant gene list yielding a total of 116 genes with available gene expression data.

### Statistical analysis

Disease free and overall survival of different TIME types were calculated by Kaplan-Meyer method and Wilcoxon test for statistical significances. Fisher’s exact test was applied to compare gene mutation frequencies between different TIME groups. Additionally, the distribution of cases with upregulated and downregulated anti-tumor immunity in *EP300* mutated and *EP300* wildtype tumors were tested for statistical significance by Fisher’s exact test. Upregulation frequency of each metabolic gene in EP300 high and low tumors was also compared by Fisher’s exact test. Unpaired two-tailed t-test was used to compare means of all upregulated genes of one metabolic phenotype in the panCancer analyses. The metabolic phenotype of cell lines after C646 treatment was compared by paired t-test. P values less than 0.05 were considered statistically significant. Statistical analyses and graph creations were performed with Prism 8 (GraphPad, San Diego, CA).

### Précis

The lysine-acetyltransferase EP300 inhibits anti-tumor immune response via modulation of tumor metabolism and might therefore represent a new target for combinatory immuno-therapeutic approaches.

## Results

### Tumor immune microenvironment subtypes in head and neck squamous cell carcinomas are associated with specific gene mutations

First, we aimed to identify gene mutations associated with different TIME subtypes within the HNSCC cohort of the TCGA database. At the time of the computational analyses (last updated 12/2018) the TCGA HNSCC cohort consisted of 530 patients with available clinical, proteomic and genomic data. We started the evaluation with the creation of three TIME subtypes based on gene expression profiles of immune-related genes as outlined in Fig. 1A:

1. An immune-activated TIME with high expressions of genes involved in cytotoxic T cell response.

2. An immune-suppressed TIME with low expression of genes involved in cytotoxic T cell response and high expression of the macrophage defining gene CD68 and S100A9, a gene with good specificity for myeloid derived suppressor cells^11^.

3. An immune-absent TIME with downregulation of all immune-related genes used for subtype stratification.

Patients with upregulation or downregulation of genes as outlined in Fig. 1A (z-Score = 0) were included into the respective TIME subtypes. In summary, this yielded 20 patients for the immune-activated group, 35 patients for the immune-suppressed group and 100 patients for the immune-absent group. The remaining 375 patients of the TCGA HNSCC cohort were excluded from further analyses, as they did not consistently fit into one of the immune gene profiles of Fig. 1A. In order to ensure clinical relevance of the gene expression-based TIME classification, we tested its impact on overall and disease free survival of the three groups (Fig. 1B). This resulted in a well-defined segmentation with best overall (p** =** 0.0619) and disease free (p** =** 0.0245) survival for the immune-activated group compared to the combination of the immune-suppressed and immune-absent group. A further comparison of clinical and tumor characteristics for the three different TIME types and the complete TCGA cohort is summarized in Table 1. The TCGA HNSCC cohort contained significantly more patients with immune-absent TIME than with immune-activated TIME (100 versus 20). The number of HPV-positive patients was significantly higher in the immune-activated group as in the other groups (45% versus 15% in the immune-suppressed and 6% in the immune absent group). Correspondingly, the immune-activated group also contained significantly more oropharyngeal squamous cell carcinomas. Furthermore patients of the immune-activated group had a lower average number of cigarette pack years. These findings of the immune-activated group reflect to a large extent the features of HPV-induced HNSCC, which are typically located in the oropharynx, frequently show a strong anti-tumor immune infiltrate and are not necessarily associated with a history of tobacco abuse^14,17^. Regarding tumor stage patients with stage IV tumors accumulated in the immune-suppressed group (75% of immune suppressed tumors versus 48% of immune-absent tumors). We also evaluated the scanned tumor tissue slides, as provided by cBioPortal and the Digital Slide Archive (DSA, www. https://cancer.digitalslidearchive.org), for histological representativeness of each TIME subset. Overall the molecular TIME types were well reflected by their histological appearances (Fig. 1C). The histological appearances matched for 18 of 20 tumors in the immune-activated group (1 tumor had an immune-absent histological appearance, 1 tumor was not evaluable). 20 of 35 tumors had matching histological appearances in the immune-suppressed group (7 tumors had an immune-activated histological appearance, 5 tumors an immune-absent histological appearance, 3 tumors were not evaluable). The immune absent group showed matching histological appearances in 46 of 100 cases (47 tumors of this group demonstrated an immune-suppressed histological appearance, 5 tumors an immune-activated histological appearance, 2 tumors were not evaluable).

**Table 1:** Patient and tumor characteristics of the complete TCGA HNSCC cohort and the three different TIME subtypes (signifcant differences compared to underlined values are marked with bold type).

	TCGA HNSCC	Immune activated	Immune suppressed	Immune absent	
Number	530	**20 (4%)**	35 (7%)	**100 (19%)**	**p** < **0.0001**
Mean age (y)	60.9	58.55	60.2	61.5	ns
Male sex	386 (73%)	18 (90%)	28 (80%)	81 (81%)	ns
HPV positive	**77 (15%)**	**9 (45%)**	**5 (14%)**	**6 (6%)**	**p** < **0.03**
**Anatomical site**					
Oropharynx	**82 (16%)**	**12 (60%)**	**4 (11%)**	**8 (8%)**	**p** < **0.0001**
Oral cavity	**320 (60%)**	**7 (35%)**	20 (57%)	**62 (62%)**	**p** < **0.05**
Hypopharynx	10 (2%)	0	2 (5.7%)	1 (1%)	ns
Larynx	117 (22%)	**1 (5%)**	9 (26%)	**29 (29%)**	**p** < **0.03**
**Stage**					
I	21 (4%)	3 (15%)	1 (3%)	4 (4%)	ns
II	**99 (19%)**	2 (10%)	**2 (6%)**	20 (20%)	**p** < **0.05**
III	107 (21%)	4 (20%)	5 (16%)	26 (26%)	ns
IV	288 (56%)	11 (55%)	**24 (75%)**	**48 (48%)**	**p** < **0.05**
**Differentiation**					
G1	64 (13%)	0 (0%)	2 (6%)	9 (9%)	ns
G2	311 (61%)	9 (53%)	21 (64%)	63 (64%)	ns
G3	125 (25%)	7 (41%)	10 (30%)	25 (26%)	ns
G4	7 (1%)	1 (6%)	0 (0%)	1 (1%)	ns
**Social history**					
Smoking (pack years)	46 (n = 299)	**27 (n** = **9)**	**50 (n** = **18)**	**48 (n** = **61)**	**p** < **0.03**
Drinks/day	3.2 (n = 222)	2.8 (n = 8)	3.8 (n = 11)	3.6 (n = 45)	ns
**Recurrence/Progression**	145 (36%, n = 398)	3 (21%, n = 14)	14 (47%, n = 30)	32 (42%, n = 77)	ns

ns = not significant.

Next, we investigated, if there were differences between the tumor mutational burden of the complete TCGA HNSCC cohort and the different TIME subtypes. The overall number of non-synonymous mutations was, however, very heterogeneous and did not significantly differ between the TIME subtypes. Consideration of HPV status did also not significantly affect the mutational count distribution (Fig. 1D).

**Figure 2:**
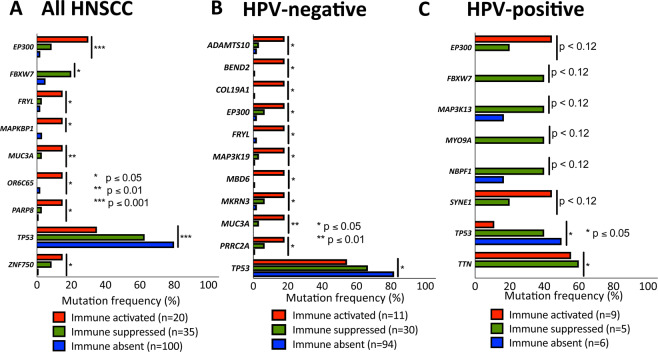
Correlation of gene mutation frequencies with TIME subtypes revealing EP300 mutation as significantly associated with an immune-activated TIME: Significant association of gene mutation frequencies with specific TIME subtypes for 9 genes of all HNSCC (**A**), 11 genes of HPV-negative HNSCC (**B**) and 8 genes of HPV-positive HNSCC. Mutations in *TP53* and *EP300* are found in all three groups.

Afterwards, we wanted to find out, if the different TIME subtypes accumulate distinct mutations. For this purpose, a list of mutated genes within the HNSCC cohort was compiled. This included only non-synonymous mutations as annotated by cBioPortal. At least 10 mutations had to be annotated for one gene across 512 profiled HNSCC samples to be included into the gene list. This represents a mutation frequency of 1.95%, to ensure feasibility of statistical work-up and clinical relevance. A total of 1519 mutated genes were included into the analysis. *TP53* had the highest mutational load with 446 mutations (mutation frequency 71.48%), followed by *TTN a*nd *FAT1 w*ith 379 (mutation frequency 41.60%) and 144 (mutation frequency 22.46%) mutations, respectively. Next, the list of 1529 mutated genes was used for a patient database query among the three different TIME subtypes (Fig. 2). Since HPV-positive HNSCC are considered a distinct molecular HNSCC entity, gene mutations were correlated for all HNSCC patients as well as for the subsets of HPV-positive and HPV-negative patients. A total of 9 and 11 mutated genes were significantly associated with one specific TIME subtype for all HNSCC and the subset of HPV-negative HNSCC, respectively. In HPV-positive HNSCC, 8 mutated genes tended to associate with specific TIME subtypes. Only 2 genes (*TP53 a*nd *TTN*), however, were found to be significantly enriched in one TIME subtype. In general, most genes were significantly associated with an immune-activated TIME, except for *TP53* mutations. Mutated *TP53* strongly correlated with an immune-absent TIME in all HNSCC subsets. This serves as a good internal control, as *TP53* mutations are known to inhibit anti-tumor immune response^18,19^. Another mutated gene, which was significantly associated with an immune-suppressed TIME in all HNSCC as well as in HPV-positive HNSCC, was *FBXW7*. Additional gene mutations with tendencies towards an immune-suppressed TIME in the HPV-positive subset were M*AP3K13*, *MYO9A* and *NBPF1*. *EP300* was together with *TP53* the only mutated gene, which was associated with one TIME subtype across all HNSCC, HPV-negative HNSCC and HPV–positive HNSCC. Its mutational status correlated with an immune-activated TIME. *EP300* is a lysine acetyltransferase similar to CREB binding protein (CBP) serving as a cofactor for the expression of numerous genes. Hence, it regulates many different processes including proliferation, cell cycle, cell differentiation and DNA damage response^20^.

### *In silico* analyses suggest lysine acetyltransferase EP300 as a panCancer modulator of anti-tumor immune response

**Figure 3:**
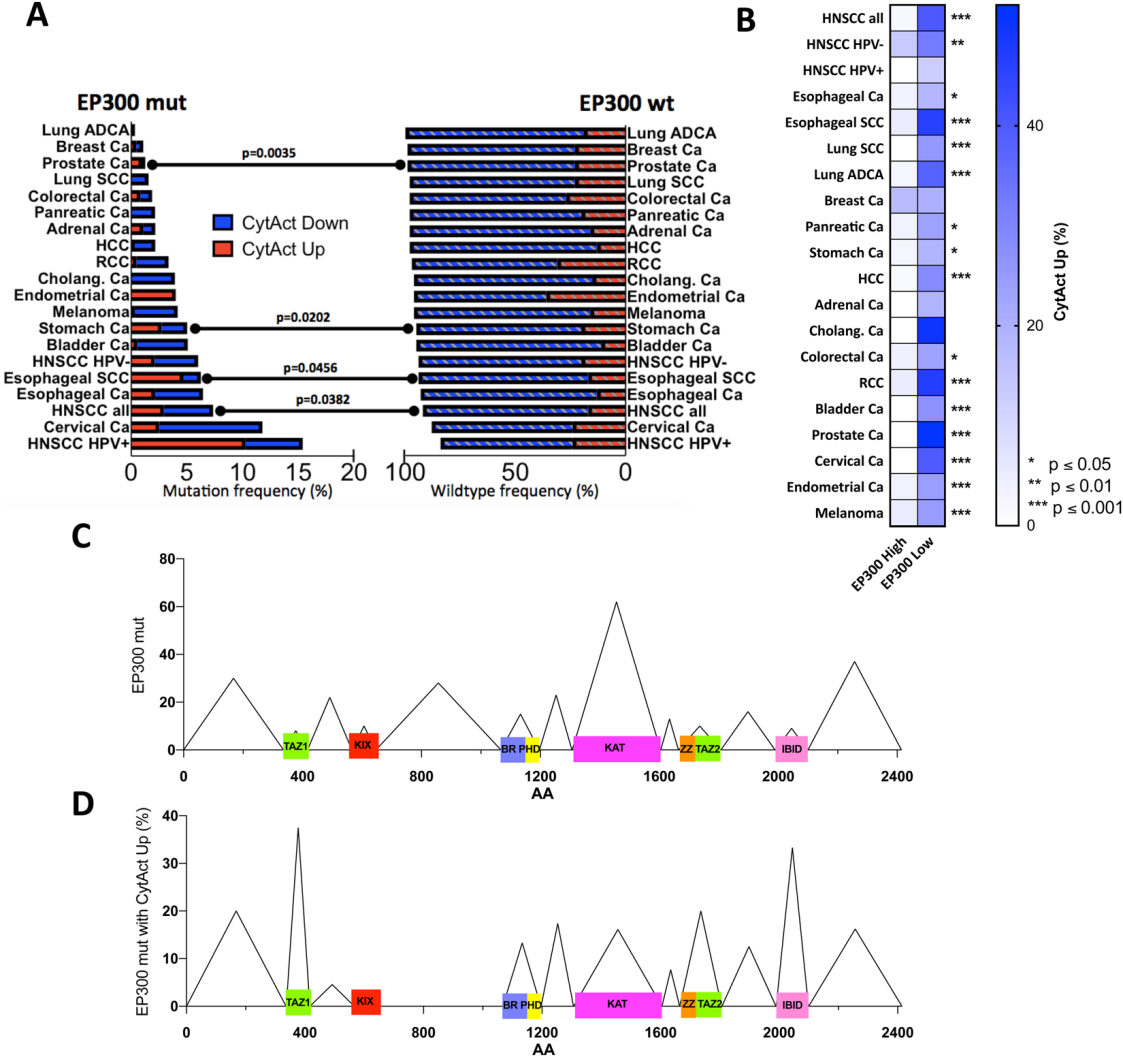
PanCancer correlation of EP300 mutation and expression status with anti-tumor immunity. (**A**) Display of *EP300* mutation frequency for 18 common solid malignancies (n = 7564) and association with cytolytic activity in comparison to *EP300* wt tumors. HNSCCs, esophageal SCC, stomach carcinomas and prostate carcinomas have a significantly higher cytolytic activity in *EP300* mut tumors. (**B**) PanCancer correlation of EP300 expression status with anti-tumor immunity for 18 solid malignancies showing a significantly higher cytolytic activity in in EP300 low tumors. (**C**) PanCancer distribution of *EP300* mutations with most mutations at the lysine acetyltransferase domaine KAT. (**D**) Percentage of *EP300* mutations with increased cytolytic activity relative to all mutations with spiking mutation counts at TAZ1 and IBID. Mut: mutated; wt: wildtype; CytAct: cytolytic activity; ADCA: adenocarcinoma; Ca: carcinoma; SCC: squamous cell carcinoma; HCC: hepatocellular carcinoma; RCC: renal clear cell carcioma; cholang: cholangiocellular; TAZ: Transcriptional adapter zinc finger; KIX: kinase inducible domain of CREB interacting domain; BR: Bromodomain; PHD: Plant homeodomain; IBID: Interferon binding domaine.

Based on the association of *EP300* mutations with an immune-activated TIME in HNSCC, we consequently, wondered, if we could confirm this in other tumor types. Most of the common solid malignancies of the TCGA database were included for the analysis: Esophageal adeno- and squamous cell carcinoma, lung adeno- and squamous cell carcinoma, breast carcinoma, gastric adenocarcinoma, cholangiocellular adenocarcinoma, pancreatic adenocarcinoma, hepatocellular carcinoma, colorectal adenocarcinoma, renal clear cell carcinoma, adrenal carcinoma, bladder carcinoma, prostate adenocarcinoma, endometrial carcinoma, cervical carcinoma, melanoma. The analysis included a total of 7564 patients with an overall *EP300* mutation prevalence of 3.1% (277 patients). The highest mutation frequency was found in HPV-associated carcinomas (15.4% of HPV-positive HNSCCs and 11.8% of uterine cervical carcinomas). Lung adenocarcinomas, breast carcinomas and prostate carcinomas had the lowest mutation frequency with 0.3%, 1.1% and 1.2%, respectively (Fig. 3A). Next, we compared the anti-tumor immunity with the mutational status of *EP300*. We used gene expression profiles of the pore-forming and proteolytic proteins perforin (PFN1) and granzyme (GZMB), to define the anti-tumor immune response. The simultaneous upregulation of PFN1 and GZMB, which are secreted by CD8** +** CTLs, has been defined as cytolytic activity by Rooney *et al*. and has been shown to correlate with neoantigen load^4^. We defined tumors with simultaneous upregulation of PRF1 and GZMB (CytAct Up, z-Score = 0) as tumors with increased anti-tumor immunity. Tumors with a simultaneous downregulation of PRF1 and GZMB (CytAct Down) were defined as tumors with downregulated anti-tumor immunity. Besides HNSCC, upregulation of cytolytic activity significantly correlated with *EP300* mutation in esophageal squamous cell carcinomas, gastric carcinomas and prostate carcinomas (Fig. 3A).

Since we hypothesized that mutation-associated functional limitations of *EP300* lead to an immune-activated TIME, we wondered, how EP300 expression correlates with anti-tumor immune response. We divided patients of all 18 solid malignancies in EP300 low and high expressers according to a z-Score = 1. This yielded a total of 916 patients with downregulated EP300 and 1181 patients with upregulated EP300. Assessing all tumor entities separately, EP300 downregulation was strongly associated with increased cytolytic activity in almost all cancer entities, except for carcinomas of the breast (Fig. 3A).

We also correlated EP300 expression with the recently described tumor immune profiles by Thorsson *et al*.^13^. These are based on TCGA data of more than 10000 tumors and stratify tumors into six different subtypes: C1 (wound-healing), C2 (Interferon-γ dominant), C3 (inflammatory), C4 (lymphocyte-depleted), C5 (immunologically quiet), C6 (TGF-β dominant). Immune subtype C2 is characterized by the highest M1/M2 macrophage polarization and the highest CD8 count. Consistent with our analyses, *EP300* mutated tumors and tumors with decreased EP300 expression showed significantly more frequently a C2 immune subtype (Suppl. Fig. 1). The C3 immune subtype, in contrast, was more frequently present in *EP300* wildtype tumors and tumors with high EP300 expression. The C3 subtype is defined by elevated Th1 and Th17 genes.

Overall distribution of *EP300* mutations within the TCGA panCancer cohort correlated with results of previous studies^20^. The histone acetyltransferase domain KAT carried the most mutations with a total of 62 non-synonymous mutations (22.2% of all *EP300* mutations). The other functional domains had low mutation frequencies ranging from 8 in the TAZ1 domain (transcriptional adapter zinc finger 1, 2.9% of all *EP300* mutations) to 15 in the BR-PHD domain (bromodomain-plant homeodomain, 5.5% of all *EP300* mutations, Fig. 3C). In contrast, the highest frequency of *EP300* mutations, which also had an upregulated cytolytic activity in the tumor, were observed in the TAZ1 domain (37.5%, n** =** 3) and the interferon binding domain IBID (33.3%, n** =** 3, Fig. 3D). On the other hand, KAT domain mutations associated with increased cytolytic activity were only found 17% of cases (n** =** 4).

### EP300 overexpression is associated with a glycolytic tumor metabolism

The association of EP300 expression with decreased anti-tumor immunity raises the question about a functional mechanism of EP300 inhibiting the tumor immune response. We and others have previously shown that tumor metabolism is an important determinant of the tumor immune microenvironment^14,15,21^. Namely a glycolytic tumor metabolism with accumulation of lactate in the tumor microenvironment leads to an inhibition of cytotoxic T cells and to an unfavorable TIME. On the other hand, an oxidative phosphorylation- (OXPHOS-) dominant tumor metabolism supports a strong anti-tumor immune response. Just recently the metabolism-modulatory functions of EP300 have been described in more detail. Among others, these studies revealed EP300’s role in promoting genes involved in glycolysis and demonstrated that EP300 inhibition leads to a disruption of glucose metabolism^22,23^. Consequently, we hypothesized that EP300 upregulation is associated with a glycolysis-dominant tumor metabolism, whereas EP300 downregulation correlates with an OXPHOS-dominant tumor metabolism.

First, we compiled lists of metabolic genes defining a glycolysis-dominant or OXPHOS-dominant metabolism. These genes were based on the publicly available collections of the glucose and OXPHOS metabolism RT^2^ Profiler PCR Array, as provided by Qiagen (Hilden, Germany).

**Figure 4:**
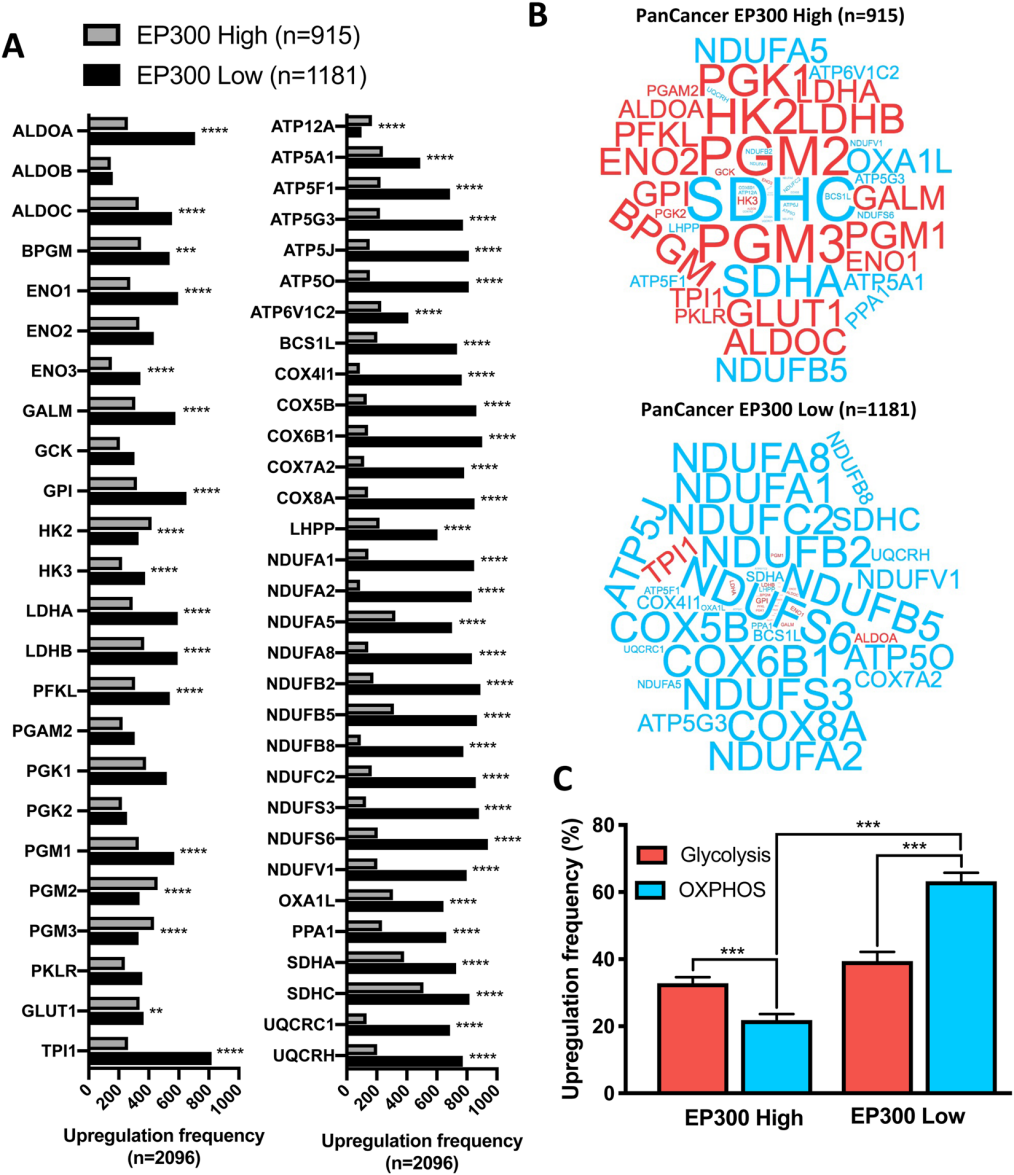
PanCancer analyses of metabolic phenotypes in relationship to EP300 expression: (**A**) Absolute upregulation frequency of 24 genes involved in glycolysis and 31 genes invovled in OXPHOS with significant upregulation of all OXPHOS genes, except for ATP12A in EP300 low tumors and significant downregulation of 14 glycolysis genes in EP300 low tumors. (**B**) PanCancer expression of metabolic genes (red: 24 genes involved in glycolysis; blue: 31 genes involved in OXPHOS) with predominance of glycolytic genes in EP300 high tumors and predominace of OXPHOS-associated genes in EP300 low tumors. Font size is relative to upregulation frequency. (**C**) Relative upregulation frequency of glycoyltic and OXPHOS-associated genes with a dominantly glycolysis-dependent metabolism in EP300 high tumors and an OXPHOS-dependent metabolism in EP300 low tumors. ** p < 0.01, ***p < 0.0001.

Next, we performed a panCancer metabolic characterization including all 18 solid malignancies mentioned in the study. Tumors were distinguished according to EP300 expression into EP300 high (n** =** 915) and EP300 low (n** =** 1181) tumors. Upregulation frequency (z-Score = 0) of all metabolic genes in both groups were calculated (Fig. 4A). 14 of 24 glycolysis-associated genes were significantly upregulated in EP300 low tumors, while 4 of 24 glycolysis-associated genes were downregulated. All OXPHOS-related genes were significantly upregulated in EP300 low tumors, except for ATP12A. Comparing the two tumor metabolism types, EP300 high tumors were dominated by an upregulation of glycolysis-associated genes, while in EP300 low tumors OXPHOS-related genes dominated (Fig. 4B). Assessing the mean upregulation frequency for all glycolysis-associated and OXPHOS-associated genes EP300 low and EP300 high tumors were significantly different with a predominantly OXPHOS-dependent metabolism for EP300 low tumors (Fig. 4C).

We also performed a panCancer comparison of the tumor metabolism in *EP300* wildtype (n** =** 4694) and *EP300* mutated (n** =** 233) tumors (Suppl. Fig. 2). Differences were only subtle, however glycolysis-related as well as OXPHOS-related genes were significantly more frequently upregulated in *EP300* mutated tumors.

### *In silico* modeling demonstrates decreased glycolytic metabolism in cell lines after EP300 inhibition

Since our previous results suggested an association of EP300 expression with a glycolytic tumor metabolism, we wondered, if EP300 inhibition leads to changes in cell metabolism. The publicly available Connectivity Map (CMap) at the Broad Institute was used to investigate the effect of EP300 inhibition on gene expression profiles of different cell lines^16^. This database includes perturbations with the small molecule C646, a widely used and specific inhibitor of EP300^24^.

**Figure 5:**
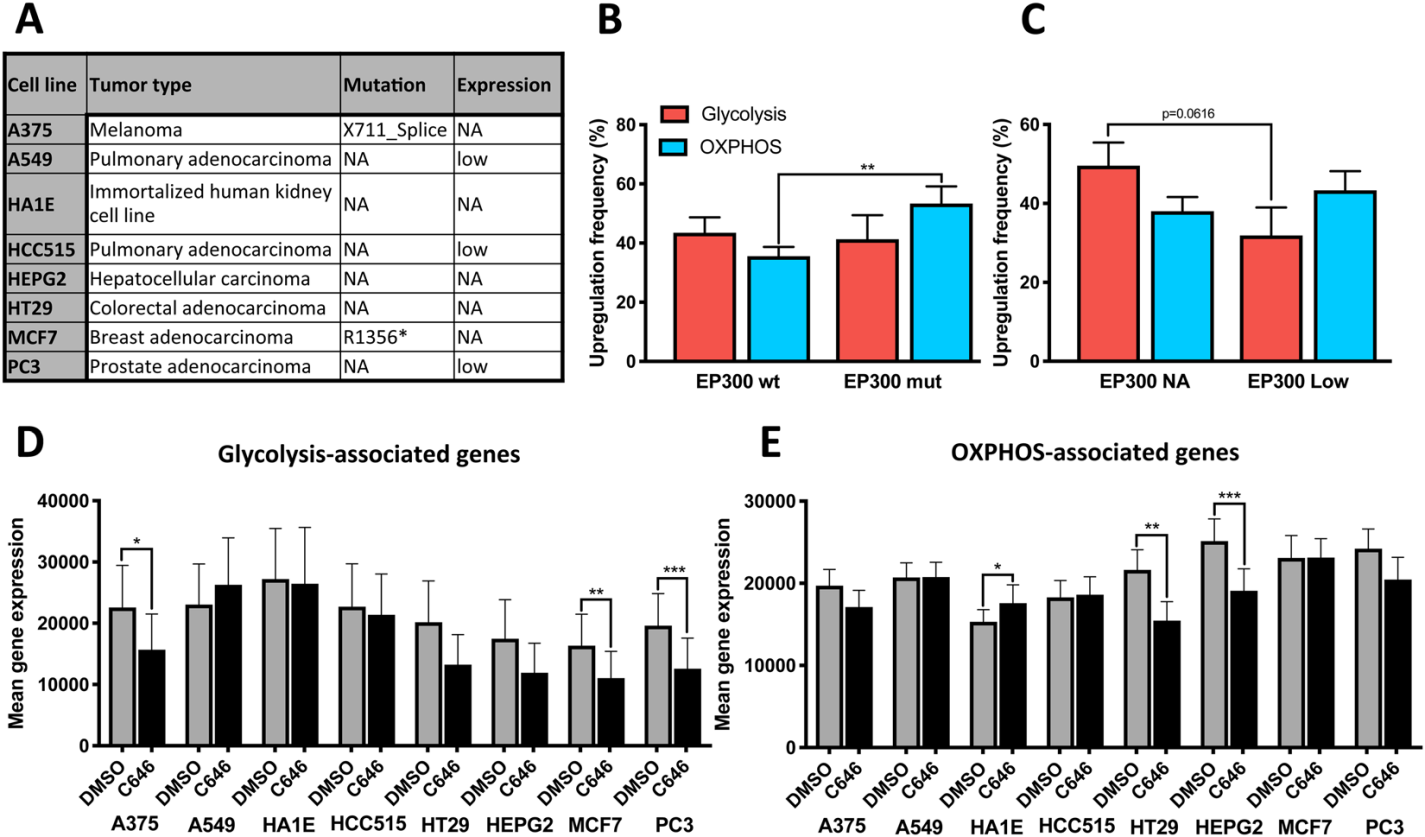
Metabolic effects of EP300 inhibition on eight cell lines via *in silico* analysis. (**A**) List of cell lines with EP300 mutation and expression status (z Score = 1). (**B,C**) Baseline metabolic characterization with signifcant upregulation of genes involved in OXPHOS in cell lines with EP300 mutation (A375, MCF7) and trends towards a decrease of genes involved in glycolysis in cell lines with low EP300 expression (A549, HCC515, PC3). (**D–E**) Changes of mean gene expression levels after treatment with the EP300 inhibitor C646 with a significant downregulation of glycolysis-associated genes (mean expression level of 52 genes) in A375, MCF7 and PC3 as well as a significant downregulation of OXPHOS-associated genes (mean expression level of 112 genes) in HT29 and HEPG2. In HAE1E OXPHOS genes significantly increased after C646 treatment. NA: No alteration; wt: wildtype; mut: mutated. *p < 0.05, **p < 0.01, ***p < 0.001.

First we assessed the EP300 mutation and expression status (z-Score = 1) of the eight cell lines, analyzed by CMap (Fig. 5A). These include the Melanoma cell line A375 and the breast adenocarcinoma cell line MCF7 with *EP300* mutations (X711_Splice and R1356*, respectively). Furthermore it includes two pulmonary adenocarcinoma cell lines A549 and HCC515 as well as one prostate carcinoma cell line (PC3) with relative downregulation of EP300 expression. Additionally, the hepatocellular carcinoma cell line (HEPG2), the colorectal adenocarcinoma cell line (HT29) and the immortalized human kidney cell line HA1E were included, which have no alterations in EP300. Upregulation of EP300 was not observed in any cell line. Metabolic characterization of the eight cell lines showed a significant upregulation of OXPHOS-associated genes for the two cell lines (A375 and MCF7) with *EP300* mutations (Fig. 5B). On the other hand, glycolysis-associated genes tended to be downregulated in the two cell lines (A549 and HCC515) with decreased EP300 expression (Fig. 5C). Next we investigated changes in cell metabolism after treatment with the EP300 inhibitor C646. 3 of 8 cell lines (A375, MCF7 and PC3) had a significantly downregulated glycolysis-associated metabolism after C646 treatment in comparison to DMSO incubation (Fig. 5D). Two additional cell lines, HT29 and HEPG2, tended to a decreased glycolytic metabolism after C646 treatment. Additionally, OXPHOS-related metabolism was significantly downregulated in HT29 and HEPG2, whereas it significantly increased in HA1E after C646 exposure.

## Discussion

Over the last years the TIME of HNSCC and other solid malignancies has been extensively studied elaborating tumors with favorable, anti-tumor or unfavorable, pro-tumor immune microenvironments^4,15,25–28^. We are, however, far away from a detailed understanding of the TIME and how we can further exploit it for best therapeutic effects. In HNSCC, for example, overall response rate to immune checkpoint inhibition is only 20%^29^. Detection of PD-L1 expression in the tumor via immunohistochemistry is currently the most widely used biomarker to determine eligibility for treatment with the PD-1 inhibitor Pembrolizumab^30^. It is approved by the FDA for 15 different tumor types. In a recent meta-analysis, it was predictive in 28.9% of cases, whereas in 53.3% it was not predictive^30^. Analyses of microsatellite instability and the tumor mutational burden are other factors, which can be used to enrich for patients likely to respond to immunotherapy. Nevertheless, we only have a limited understanding, which patient will benefit from immunotherapy and how we can expand the efficacy of checkpoint inhibition for patients with an immunotherapeutic ineligible TIME.

This study aimed to extend our knowledge on the association of the tumor genome and specific TIME types, in order to detect new biomarkers and therapeutic targets for immunotherapies. We started with HNSCC, as this is a tumor entity with a relatively high mutational burden. Additionally, the TIME of HNSCC is known to be associated with treatment response, but HNSCC often show a limited response to immunotherapeutic approaches^31,32^. In the first part of the study, we defined 3 different, clinically-relevant TIME subtypes in HNSCC based on immune-related gene expressions. In the next step we could connect about 8–11 altered genes (depending on the HPV status) to a specific TIME type. The association of TIME subtypes with mutated genes appeared to depend on the HPV status (HPV-positive or HPV-negative) of the tumor underlining the differences of these two distinct molecular entities. Furthermore, the diverging gene list between HPV-negative and HPV-positive HNSCC demonstrate that it is hardly possible to connect the HNSCC TIME subtypes to specific genetic alterations. It is rather a multifactorial combination of tumor genetic properties and host immune defense. Additionally, it is important to mention that we probably discovered only a limited number of genes with potential immune-oncological relevance. Our analysis approach included 155 of 530 patients of the HNSCC TCGA cohort, who fit into one of the immune subgroups. The strict definition of our immune subgroups prevented bigger cohorts for the screening approach. But this made it, on the other hand, more likely that genes with a significant association with one of the subgroups have a relevant connection to the TIME.

*EP300* was besides *TP53*, the only mutated gene, which was associated with a specific TIME in HPV-negative as well as HPV-positive HNSCC. We therefore focused on EP300 for further analyses, as it has not drawn much attention to its role in tumor immune activity. EP300 is a lysine acetyltransferase similar to CREB-binding protein (CBP). It regulates the cell cycle as a transcriptional coactivator, e. g. of E2F, and via histone modification through acetylation. EP300 and CBP bind to more than 16000 genes in human cells leading to a wide range of functions in healthy and tumor tissue^33,34^. On the one hand, EP300 may have tumor suppressive functions by promoting other tumor suppressors as *TP53*, *RB1* or *BRCA1*^20^. On the other hand, EP300 overexpression has been described to be associated with poorer outcome and higher aggressiveness in several tumors including HNSCC and cutaneous squamous cell carcinomas, colorectal cancers and hepatocellular carcinoma^35–38^. It is, however, still unclear, how EP300 overexpression promotes aggressiveness of tumors. So far EP300 has rarely been associated with tumor immunity. Two studies elaborated the role of EP300 for regulatory T cell differentiation and function^39,40^. More importantly, Liu *et al*. showed that EP300 inhibition reduced tumor growth in wild type, but not immune-deficient mice and increased tumor infiltration as well as activity of cytotoxic T cells and decreased Tregs^40^.

Our *in silico* analyses indicate a tumor promoting effect of EP300 via immunosuppression. Based on the panCancer analyses of our study we were able to further dissect a potential functional background of EP300-related tumor immune suppression. We observed an immune-activated TIME in several *EP300* mutated as well as most EP300 downregulated tumors across many different solid malignancies. Evaluation of the location of *EP300* mutations associated with increased anti-tumor immunity demonstrated that there is no specific *EP300* mutation responsible for tumor immune-modulatory effects. Most *EP300* mutations are located in the histone modifying KAT domain. Focusing on *EP300* mutations with anti-tumor immunity association, we detected mutation spikes in the TAZ1 and IBID domain. While TAZ1 binds among others the transcriptional factor HIF1α, which is the key factor of tumor metabolism, IBID binds IRF-3, which is involved in innate immunity control^41^. This gives a first suggestion, that the TAZ1 and IBID domain might play functional roles in modulating the TIME.

Another exciting incidental finding of our study is the association of *EP300* mutations with HPV infection. Just recently an association of *EP300* mutations and intragenic *EP300* deletions were discovered for HPV-positive oropharyngeal squamous cell carcinomas in two independent studies^42,43^. The HPV proteins E2 and E7 have been described to bind to EP300^42,44–47^. Functional analyses suggest that EP300 is necessary to initiate E2 induced viral transcription and replication. Recruitment of EP300 together with retinoblastoma protein by E7 is suspected to be a key factor in retinoblastoma protein dependent disruption of the cell cycle control. At this point further assessments of the location of HPV-associated *EP300* mutations and functional *in vitro* studies would, however, be needed to explain the high *EP300* mutation frequency in HPV-related cancer.

Recent studies have shown that EP300 is a promoter of glycolysis and inhibition of EP300 leads to inhibition of glucose metabolism^22,23^. Tumor cell glucose metabolism, however, is known to exert inhibitory effects on T cell proliferation, tumor infiltration by T cells and T cell guided anti-tumor immune response^48^. In our panCancer metabolic studies we could demonstrate that tumors with high EP300 expression have a predominantly glycolysis-associated metabolism. Tumors with low EP300 expression, on the contrary, had an OXPHOS-dominated metabolism. *In silico* analyses of cell lines treated with the EP300 inhibitor C646 underlined this observation. We observed changes of metabolism-related genes in 6 of 8 cell lines after C646 treatment. Glycolysis-associated genes significantly decreased in 3 cell lines and tended to decrease in 3 additional cell lines. OXPHOS-associated genes decreased in 2 cell lines and increased in 1 cell line. Taken together, we see an inhibition of tumor cell metabolism in cell lines already after inhibition of EP300 for a short period of time (24 hours). Our cell line studies indicate that changes in the tumor metabolism could be the link between EP300 expression and tumor immune evasion.

We think that our results support testing the inclusion of EP300 inhibitors into immunotherapeutic approaches. By now there exist several EP300 and EP300/CBP specific inhibitors, which have shown promising tumor inhibiting effects *in vitro* and in preclinical studies^49–53^. The anti-tumor effects of the selective EP300 and CBP inhibitor CCSS1477 are currently investigated in a clinical trial of metastatic castration resistant prostate cancer and other advanced solid tumors (NCT03568656).

This *in silico* study allows the correlation of EP300 with different tumor characteristics over a large number of tumors and cancer entities. Thereby our results give exciting perspectives, how to expedite research focuses for EP300 and how to integrate it into preclinical and clinical therapeutic approaches. Preclinical experiments investigating the effect of EP300 inhibition on the TIME, for example in syngeneic mouse models, would constitute a next step to assess EP300 as an immune-oncological target.

## Supplementary information

Supplementary figure 1 and 2.

## References

[1] Donnem T (2016). Strategies for clinical implementation of TNM-Immunoscore in resected nonsmall-cell lung cancer. Ann. Oncol. Off. J. Eur. Soc. Med. Oncol..

[2] Turan T (2018). Immune oncology, immune responsiveness and the theory of everything. J. Immunother. Cancer.

[3] Bindea G (2013). Spatiotemporal Dynamics of Intratumoral Immune Cells Reveal the Immune Landscape in Human Cancer. Immunity.

[4] Rooney MS, Shukla SA, Wu CJ, Getz G, Hacohen N (2015). Molecular and genetic properties of tumors associated with local immune cytolytic activity. Cell.

[5] Chimal-Ramírez, G. K., Espinoza-Sánchez, N. A. & Fuentes-Pananá, E. M. Protumor Activities of the Immune Response: Insights in the Mechanisms of Immunological Shift, Oncotraining, and Oncopromotion. *J. Oncol*. **2013** (2013). 10.1155/2013/835956 PMC3612474 23577028

[6] Blankenstein T, Coulie PG, Gilboa E, Jaffee EM (2012). The determinants of tumour immunogenicity. Nat. Rev. Cancer.

[7] Goodman AM (2017). Tumor Mutational Burden as an Independent Predictor of Response to Immunotherapy in Diverse Cancers. Mol. Cancer Ther..

[8] Wang, S., He, Z., Wang, X., Li, H. & Liu, X.-S. Antigen presentation and tumor immunogenicity in cancer immunotherapy response prediction. *eLife* **8** (2019). 10.7554/eLife.49020 PMC6879305 31767055

[9] Lawrence MS (2013). Mutational heterogeneity in cancer and the search for new cancer genes. Nature.

[10] Hanna, G. J. *et al*. Frameshift events predict anti-PD-1/L1 response in head and neck cancer. *JCI Insight* **3** (2018). 10.1172/jci.insight.98811 PMC5916245 29467336

[11] Zhao F (2012). S100A9 a new marker for monocytic human myeloid-derived suppressor cells. Immunology.

[12] Gutman DA (2017). The Digital Slide Archive: A Software Platform for Management, Integration, and Analysis of Histology for Cancer Research. Cancer Res..

[13] Thorsson V (2019). The Immune Landscape of Cancer. Immunity.

[14] Krupar R (2014). Immunologic and metabolic characteristics of HPV-negative and HPV-positive head and neck squamous cell carcinomas are strikingly different. Virchows Arch. Int. J. Pathol..

[15] Krupar R (2018). Immunometabolic Determinants of Chemoradiotherapy Response and Survival in Head and Neck Squamous Cell Carcinoma. Am. J. Pathol..

[16] Subramanian A (2017). A Next Generation Connectivity Map: L1000 Platform and the First 1,000,000 Profiles. Cell.

[17] Krupar R (2013). Comparison of HPV prevalence in HNSCC patients with regard to regional and socioeconomic factors. Eur. Arch. Otorhinolaryngol..

[18] Cooks T, Harris CC, Oren M (2014). Caught in the cross fire: p53 in inflammation. Carcinogenesis.

[19] Guo G, Yu M, Xiao W, Celis E, Cui Y (2017). Local Activation of p53 in the Tumor Microenvironment Overcomes Immune Suppression and Enhances Antitumor Immunity. Cancer Res..

[20] Attar, N. & Kurdistani, S. K. Exploitation of EP300 and CREBBP Lysine Acetyltransferases by Cancer. *Cold Spring Harb. Perspect. Med*. **7** (2017). 10.1101/cshperspect.a026534 PMC5334244 27881443

[21] Fischer K (2007). Inhibitory effect of tumor cell-derived lactic acid on human T cells. Blood.

[22] Huang H (2018). EP300-Mediated Lysine 2-Hydroxyisobutyrylation Regulates Glycolysis. Mol. Cell.

[23] He H (2017). Selective p300 inhibitor C646 inhibited HPV E6-E7 genes, altered glucose metabolism and induced apoptosis in cervical cancer cells. Eur. J. Pharmacol..

[24] Bowers EM (2010). Virtual ligand screening of the p300/CBP histone acetyltransferase: identification of a selective small molecule inhibitor. Chem. Biol..

[25] Chen Y-P (2017). Genomic Analysis of Tumor Microenvironment Immune Types across 14 Solid Cancer Types: Immunotherapeutic Implications. Theranostics.

[26] Cao B, Wang Q, Zhang H, Zhu G, Lang J (2018). Two immune-enhanced molecular subtypes differ in inflammation, checkpoint signaling and outcome of advanced head and neck squamous cell carcinoma. Oncoimmunology.

[27] Lechner A (2017). Characterization of tumor-associated T-lymphocyte subsets and immune checkpoint molecules in head and neck squamous cell carcinoma. Oncotarget.

[28] Fang J (2017). Prognostic significance of tumor infiltrating immune cells in oral squamous cell carcinoma. BMC Cancer.

[29] Dogan V, Rieckmann T, Münscher A, Busch C-J (2018). Current studies of immunotherapy in head and neck cancer. *Clin. Otolaryngol. Off. J. ENT-UK Off*. J. Neth. Soc. Oto-Rhino-Laryngol. Cervico-Facial Surg..

[30] Davis AA, Patel VG (2019). The role of PD-L1 expression as a predictive biomarker: an analysis of all US Food and Drug Administration (FDA) approvals of immune checkpoint inhibitors. J. Immunother. Cancer.

[31] Cohen, E. E. W. *et al*. The Society for Immunotherapy of Cancer consensus statement on immunotherapy for the treatment of squamous cell carcinoma of the head and neck (HNSCC). *J. Immunother. Cancer* **7** (2019). 10.1186/s40425-019-0662-5 PMC6632213 31307547

[32] Zhang X-M (2018). Prognostic and predictive values of immune infiltrate in patients with head and neck squamous cell carcinoma. Hum. Pathol..

[33] Ramos YFM (2010). Genome-wide assessment of differential roles for p300 and CBP in transcription regulation. Nucleic Acids Res..

[34] Smith JL (2004). Kinetic profiles of p300 occupancy *in vivo* predict common features of promoter structure and coactivator recruitment. Proc. Natl. Acad. Sci. USA.

[35] Chen M-K (2015). Overexpression of p300 correlates with poor prognosis in patients with cutaneous squamous cell carcinoma. Br. J. Dermatol..

[36] Cho Y-A (2014). The role of p300 in the tumor progression of oral squamous cell carcinoma. J. Oral Pathol. Med..

[37] Kowalczyk AE (2017). Expression of the EP300, TP53 and BAX genes in colorectal cancer: Correlations with clinicopathological parameters and survival. Oncol. Rep..

[38] Li M (2011). High expression of transcriptional coactivator p300 correlates with aggressive features and poor prognosis of hepatocellular carcinoma. J. Transl. Med..

[39] Ghosh S (2016). Regulatory T Cell Modulation by CBP/EP300 Bromodomain Inhibition. J. Biol. Chem..

[40] Liu Y (2013). Inhibition of p300 impairs Foxp3^+^ T regulatory cell function and promotes antitumor immunity. Nat. Med..

[41] Wang F, Marshall CB, Ikura M (2013). Transcriptional/epigenetic regulator CBP/p300 in tumorigenesis: structural and functional versatility in target recognition. Cell. Mol. Life Sci..

[42] Haft S (2019). Mutation of chromatin regulators and focal hotspot alterations characterize human papillomavirus-positive oropharyngeal squamous cell carcinoma. Cancer.

[43] Dogan, S. *et al*. Identification of prognostic molecular biomarkers in 157 HPV-positive and HPV-negative squamous cell carcinomas of the oropharynx. *Int. J. Cancer*, 10.1002/ijc.32412 (2019). 10.1002/ijc.32412 PMC7595146 31093971

[44] Thomas, Y. & Androphy, E. J. Acetylation of E2 by P300 Mediates Topoisomerase Entry at the Papillomavirus Replicon. *J. Virol*. **93** (2019). 10.1128/JVI.02224-18 PMC6430547 30651357

[45] Jansma AL (2014). The high-risk HPV16 E7 oncoprotein mediates interaction between the transcriptional coactivator CBP and the retinoblastoma protein pRb. J. Mol. Biol..

[46] Fera D, Marmorstein R (2012). Different regions of the HPV-E7 and Ad-E1A viral oncoproteins bind competitively but through distinct mechanisms to the CH1 transactivation domain of p300. Biochemistry.

[47] Bernat A, Avvakumov N, Mymryk JS, Banks L (2003). Interaction between the HPV E7 oncoprotein and the transcriptional coactivator p300. Oncogene.

[48] Singer, K., Cheng, W.-C., Kreutz, M., Ho, P.-C. & Siska, P. J. Immunometabolism in cancer at a glance. *Dis. Model. Mech*. **11** (2018). 10.1242/dmm.034272 PMC6124550 30076128

[49] Theodoulou NH, Tomkinson NC, Prinjha RK, Humphreys PG (2016). Clinical progress and pharmacology of small molecule bromodomain inhibitors. Curr. Opin. Chem. Biol..

[50] Lasko LM (2017). Discovery of a selective catalytic p300/CBP inhibitor that targets lineage-specific tumours. Nature.

[51] Pegg N (2017). Characterisation of CCS1477: A novel small molecule inhibitor of p300/CBP for the treatment of castration resistant prostate cancer. J. Clin. Oncol..

[52] Yan Y (2019). Activity of NEO2734, a novel dual inhibitor of both BET and CBP-P300, in SPOP-mutated prostate cancer. J. Clin. Oncol..

[53] Wang Y-M (2017). Histone acetyltransferase p300/CBP inhibitor C646 blocks the survival and invasion pathways of gastric cancer cell lines. Int. J. Oncol..

