# Supplementary material for: In silico analysis reveals EP300 as a panCancer inhibitor of anti-tumor immune response via metabolic modulation
Source: Sci Rep. 2020 Jun 10;10:9389. doi: 10.1038/s41598-020-66329-7 (PMC7287052; doi:10.1038/s41598-020-66329-7)
Supplement: Supplementary file 1 — Supplementary figure 1 and 2. [file 41598_2020_66329_MOESM1_ESM.pdf]

***In silico* analysis reveals EP300 as a panCancer inhibitor of anti-tumor immune response via metabolic modulation.**

Rosemarie Krupar[1,2,+,*]; Christian Watermann[1,+]; Christian Idel[3]; Julika Ribbat-Idel[2]; Anne Offermann[2]; Helen Pasternack[2]; Jutta Kirfel[2]; Andrew G. Sikora[4], Sven Perner[1,2]

[1]Research Center Borstel, Leibniz Lung Center, Borstel, Germany

[2]Pathology of the University Hospital Schleswig-Holstein, Campus Luebeck, Germany

[3]Department of Otorhinolaryngology, University Hospital Schleswig-Holstein, Luebeck, Germany

[4]Department of Otorhinolarygology – Head and Neck Surgery, Baylor College of Medicine, Houston, USA

[+]These authors contributed equally to the work

***Corresponding author:** Rosemarie Krupar, Research Center Borstel, Leibniz Lung Center, Parkallee 3A, 23845, Borstel, Germany, Email: rkrupar@fz-borstel.de, Phone: +49-4537-188-6290, Fax: +49-04537-188-2290

Supplementary figures

Suppl. figure 1

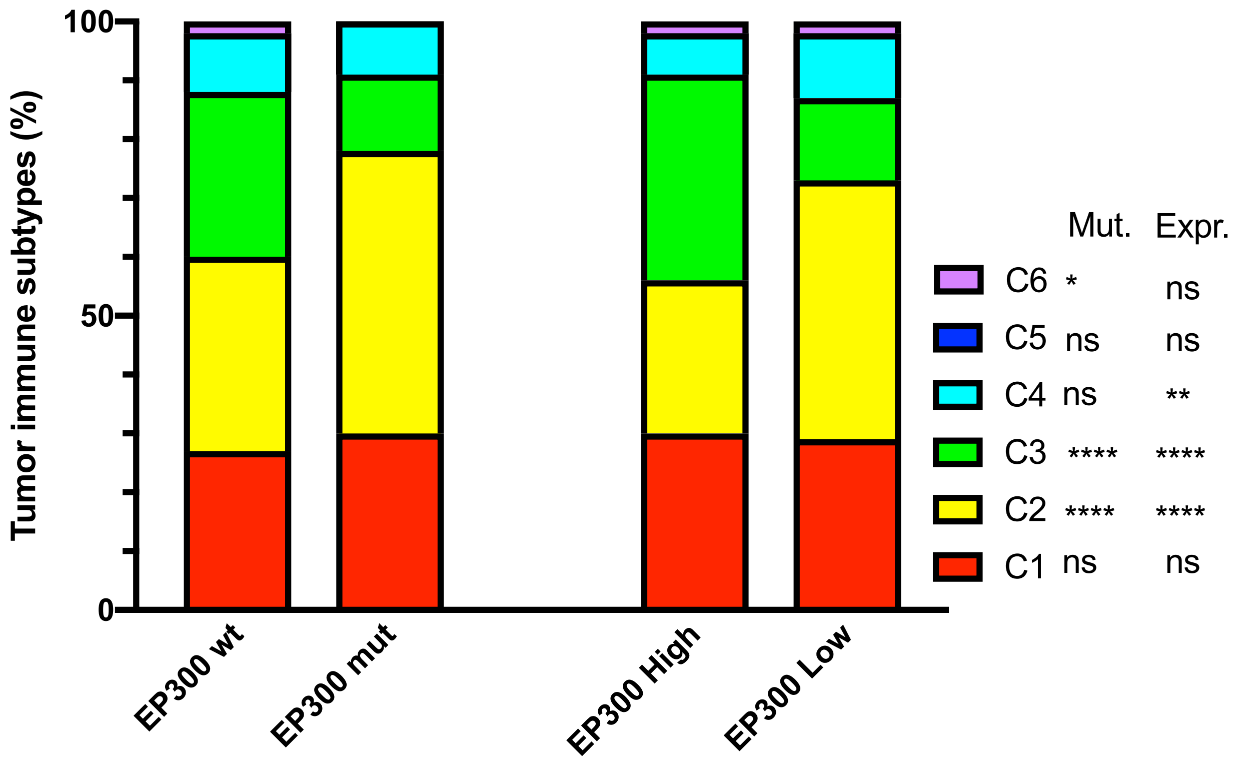

Suppl. figure 2

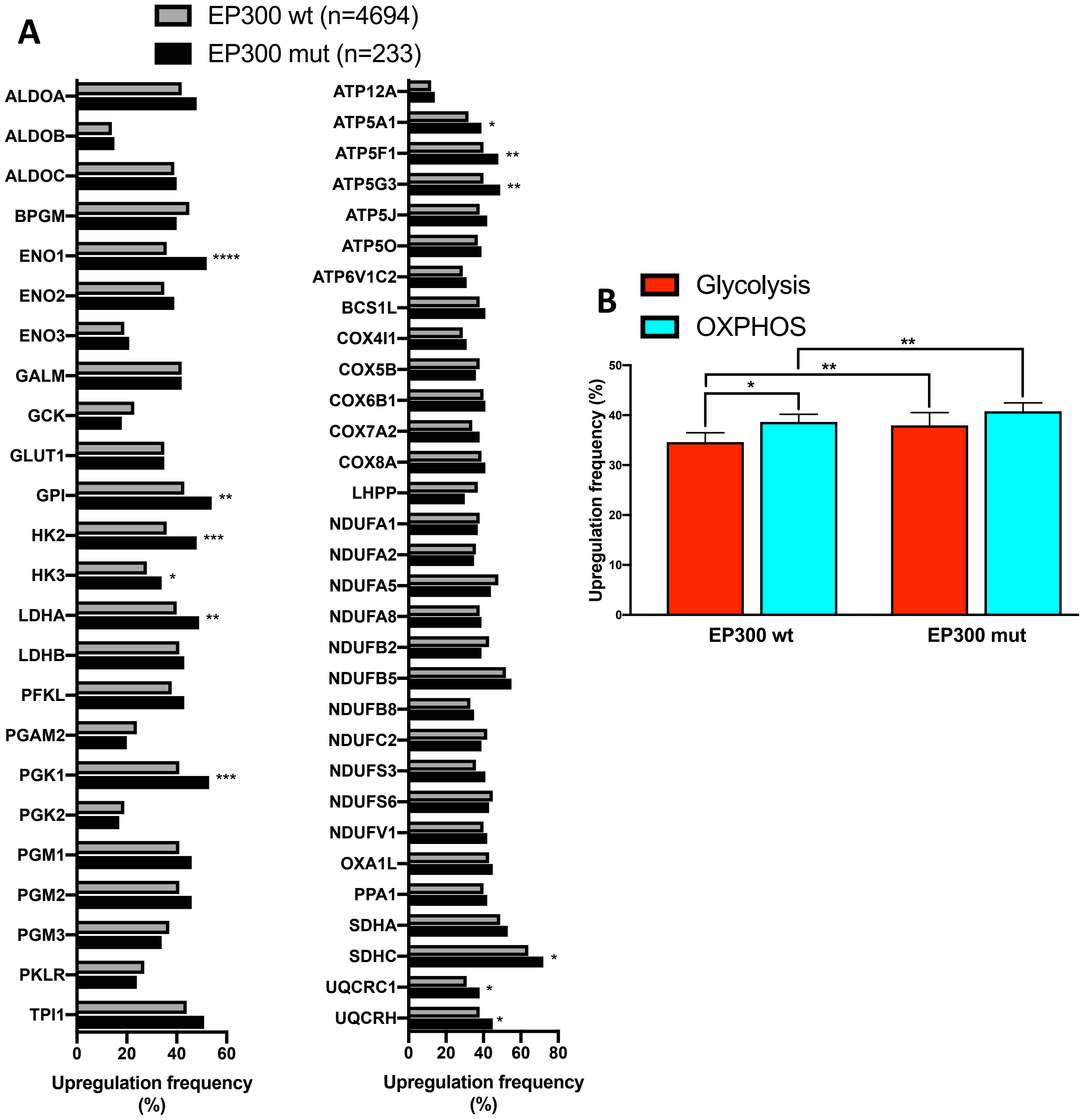

<u>Figure legend supplementary figures:</u>

Suppl. figure 1: Association of EP300 expression and mutation status with the tumor immune landscape across 18 solid malignancies. The distribution of the six immune profiles (C1 – C6), defined by Thorsson *et al.*, were compared between tumors with *EP300* wt and *EP300* mut status as well as EP300 high and low expression. The Interferon-γ dominant immune subtype C2 was significantly more frequent in *EP300* mut and EP300 low tumors. In contrast the inflammatory subtype C3 was more often found in *EP300* wt and EP300 high expressing tumors. Additionally EP300 low tumors had a higher number of C4 subtypes (lymphocyte depleted). C1: wound-healing, C2: Interferon-γ dominant, C3: inflammatory, C4: lymphocyte depleted, C5: immunologically quiet, C6: TGF-β dominant. wt: wildtype; mut: mutated; Mut: Mutation; Expr.: Expression; ns: not siginficant. *: $p<0.05$, **: $p<0.01$, ***: $P<0.001$, ****: $p<0.0001$.

Suppl. figure 2: PanCancer analyses of metabolic phenotypes in relationship to *EP300* mutation status: A) Upregulation frequency relative to number of analyzed tumors for *EP300* wt (n=4694) and *EP300* mut (n=233) displaying 24 genes involved in glycolysis and 31 genes invovled in OXPHOS. **B)** Relative upregulation frequency of glycolysis- and OXPHOS-associated genes with a subtle, but significant higher glycolysis- and OXPHOS-dependent metabolism in *EP300* mut tumors. *: $p<0.05$, **: $p<0.01$, ***: $P<0.001$, ****: $p<0.0001$.
